# Trans dermal estrogen (oestrogel) for endometrial preparation in freeze embryo transfer cycle: An RCT

**Published:** 2018-01

**Authors:** Ensieh Shahrokh Tehraninejad, Roya Kabodmehri, Batol Hosein Rashidi, Mina Jafarabadi, Fateme Keikha, Masomeh Masomi, Fedieh Hagholahi

**Affiliations:** *Health Reproductive Research Center, Imam Khomeini Hospital, Tehran University of Medical Sciences, Tehran, Iran.*

**Keywords:** FET cycle, Transdermal estrogen, Estradiol valerate GnRH analogue

## Abstract

**Background:**

Estrogen and progesterone are two crucial factors for endometrial preparation in frozen embryo transfer (FET) cycles. Studies assessing different forms of estradiol in FET have published already but literature lacks enough surveys on transdermal estrogen application in reproductive medicine.

**Objective:**

To investigate the effects of trans dermal estrogen (Oestrogel) on pregnancy rates in patients that candidate for FET cycle.

**Materials and Methods:**

In this randomized clinical trial, 100 women undergoing FET cycles referred to Imam Khomeeini Hospital were enrolled in two groups, randomly. Group I received 8 mg/day estradiol valerate (E2 tablet) orally and group II were treated with 6 mg/day transdermal oestrogel gel after suppression with gonadotropin releasing hormone agonist. In both groups medication were started in the first day of menstruation cycle and continued until endometrial thickness reached 8 mm. Pregnancy rates (chemical, clinical, and ongoing), abortion rate, live birth rate, and frequency of complications were compared between two groups.

**Results:**

Chemical and clinical pregnancy rates were not significantly different between two groups (p=0.384). The abortion rate was significantly lower in group II than group I (p=0.035). Ongoing pregnancy and the live birth rates were significantly higher in group II (p=0.035). The rate of complication was not different in two groups.

**Conclusion:**

Oestrogel seems to enhance ongoing pregnancy and live birth rates in comparison to estradiol valerate tablet.

## Introduction

Approximately 25% of all childbirths in assisted reproductive treatment cycles are achieved by frozen thawed embryo transfer cycle (1, 2). Considering frozen embryo replaced through the window of endometrial receptivity and synchronization between embryo and endometrium is important (1). This synchronization between embryonic and endometrial development is a vital factor for satisfying outcome (3). 

Frozen thawed embryo transfer was used for different groups of patients including patients with no appropriate ovarian function (4, 5) or patients with ovarian function and no transfer of fresh embryo because of genetic abnormality, hyper stimulation syndrome or egg donation (4, 6-8).

Several different protocols are available for preparation of endometrium. In patients with ovarian function, there are two main protocols: natural monitored cycle and artificial cycle (9). In patients with ovarian function who need oocyte donation (previous poor oocyte retrieval or genetic abnormality), synchronization between donor and recipient is difficult (7). The uncontrolled luteinizing hormone surge in the recipient makes it mandatory to have a close monitoring of patient with serum estradiol and luteinizing hormone assay in a natural cycle performed without any drug (1). The artificial cycle was performed with two methods: with or without gonadotropin releasing hormone agonist (GnRH-a) (10). In GnRH-a method, agonist was used for 14 days or more until assurance of gonadal suppression and then hormonal replacement therapy was performed (4, 11). In protocol without agonist, down-regulation of hypothalamus axis is achieved with administration of 17B estradiol and progesterone (4, 12-14). For estrogen replacement therapy, there are several ways of administration including oral tablets, transdermal patch, the vaginal ring and transdermal gel (4).

Tablet: Oral estrogen is converted to oestrone in the intestinal milieu (15), and oestrone is converted to estradiol in liver (4, 12). With this process serum, estradiol level was reduced around 30% (4, 9, 12). Estradiol valerate and micronized form of estradiol are usual forms used in hormonal replacement therapy (16).

Transdermal method: In the use of transdermal patch, there is not the first passage of liver and serum level of estradiol similar to the oral form. In this method, the implant does not affect the lipoprotein and clotting factor (4, 17). The use of trans dermal form has been shown not to be associated with an increase of mammographic density in the breast. Use of estradiol gel is very safe in women with increased risk of Vein thromboembolism. Use of estradiol gel does not impair lipid and clotting factor balances (18). 

The main objective of the present study was to evaluate the use of trans dermal estradiol and compare it with estradiol tablet in preparation of endometrium in frozen embryo transfer (FET) cycles.

## Materials and methods

One hundred volunteers for FET cycle (due to premature ovarian failure, ovarian hyper stimulation syndrome (OHSS) or other reasons) were enrolled in this prospective, randomized, single-blind clinical trial. The study was carried out in Reproductive Health Research Center, Tehran University of Medical Sciences between September 2015 and November 2016. Participants have been allocated by a nurse to use either estradiol valerate tablet (Group I, n=50) or trans dermal gel (Group II, n=50) according to a computer-generated randomization list. Physicians were not aware of patient allocation. Patients have participated in the study once only. Exclusion criteria were included; allergy to agonist and transfer of embryo in a fresh cycle. 

All participants received GnRH agonist (Decapeptyl, Ferring, Switzerland) 0.1 mg subcutaneously from the 21^st^ day of the cycle and it was continued for at least 14 days. In the first day of menstrual cycle suppression of ovary have been confirmed by ultrasonography. In the group I estradiol valerate tablet (8 mg/day) (Progynova, Schering, Berlin, Germany) and in group II [topical estradiol gel] (6 mg/day) (Oestrogel, 17B estradiol 0.06% gel, Besins, France) was started from the 1^st^ day of menstruation. One week after the start of estradiol, ultrasonography was performed for estimating endometrial thickness and repeated if necessary. 

On the 13th day of menstrual cycle, the second ultrasound was performed and the thickness of endometrium was estimated. If endometrial thickness was more than 8 mm, based on embryo’s age, progesterone (Cyclogest, 400 mg, Cox Pharmaceuticals, Barnstaple, UK,) was given for four to six days before embryo transfer. The transfer was performed in operation room with cook catheter (COOK Medical, Ireland, Ltd.) under sterile condition. The primary outcome was the biochemical pregnancy that was defined as a serum β human chorionic gonadotropin level greater than 5 IU/l 14 days after embryo transfer. 

Secondary outcomes included clinical pregnancy, Abortion rate, ongoing pregnancy rate, and live birth rate. [Clinical pregnancy was confirmed by ultrasonography by observing a pregnancy sac at 7 wk of gestation]. Abortion was defined as a non-viable intrauterine pregnancy after 12 wk of gestation. Ongoing pregnancy was defined as a viable intrauterine pregnancy after 12 wk of gestation.

**Ethical consideration**

The study was approved by the Ethics Committee Review Board of [Tehran University of Medical Sciences Ethics Committee Review Board]. The written informed consent was obtained from all participants (number: IR.TUMS.VCR.REC. 1395.1312). 

**Statistical analysis**

Statistical analysis was done according to the intention-to-treat basis with SPSS software (Statistical Package for the Social Sciences, version 19.0, SPSS Inc, Chicago, Illinois, USA) that included all patients who received estradiol and progesterone. For quantitative data with a normal distribution, we used independent sample t-test and data were reported as a mean standard deviation. For qualitative data, we used Chi square and Fischer-exact test. The level of significance of p<0.05 was used for all evaluation.

## Results

From September 2015 to November 2016 a total of 100 women were randomly assigned to undergo FET cycle [No patients were exclude to follow-up. The baseline characteristics were similar in the two study groups.]. [Both groups were matched for age, body mass index (BMI), endometrial thickness and duration of endometrial preparation before embryo transfer, and duration of infertility (Table I)]. Clinical characteristics such as causes of infertility and FET cycle and history of previous pregnancy and in vitro fertilization (IVF) are shown in table II. The cause of infertility was not significantly different between two groups. In group II causes of FET were the previous OHSS in 42%, egg and embryo donation in 19%, and other causes in 10%. In group, I the causes of FET were OHSS in 19%, egg, embryo donation in 16%, and other causes in 15%. In both groups, 37% of participants had no history of child bearing. In the term of IVF success history, 4 women in group I (8%) and 5 women in the group II (10%) had one successful experience which was not significantly different between two groups. As compared with patients in group I, group II had a significantly higher rate of ongoing pregnancy and live birth rate [(91% [11 of 12] vs. 50 [4 of 12]; rate ratio, 0.035). The biochemical and clinical pregnancy rates were similar between two groups and did not show significant difference. 24% of participants in group II and 16% of women in groups I had a positive [chemical and clinical pregnancy rate. The abortion rate were [significantly lower in group II (8.3% vs. 50%). None of the group showed any complication.

In order to remove the effects of confounding factors (endometriosis, BMI, number of the transferred embryo, grade and quality of embryo) on positive pregnancy test, logistic regression analysis was used. The intervention (tablet or gel) had no significant effect on pregnancy rate (p=0.317) and after eliminating the confounding effects still it was not significantly different between two groups (p=0.384).

Patient Satisfaction Survey: 1- Patients were totally satisfied medication. 2- Patients were satisfied to an acceptable level of drug.3- Patients were not satisfied at all of medicine. According to visual analog scale, based on the Patient Satisfaction Survey which was performed at the end of the treatment, 30% of patients in group II were totally satisfied, 10% were satisfied to an acceptable level and 10% were not satisfied at all. In the group I; 40% of women were totally satisfied and 10% were satisfied to an acceptable level.

**Table I:** Demographic and ultrasound characteristic factors in two groups (n=50)

	**Group I (Tablet group)**	**Group II (Gel group)**	**p-value**
Age (Yr)	31.20 ± 6.57	33.00 ± 6.60	0.176
BMI (kg/m^2^)	25.11 ± 4.23	25.86 ± 7.36	0.534
Duration of endometrial preparation day]	16.7 ± 4.09	17.50 ± 4.56	0.359
Duration of infertility (yr)	6.49 ± 5.23	6.28 ± 4.57	0.831
Endometrial thickness (mm)	8.63 ± 1.21	8.52 ± 1.46	0.701

All data presented as mean±S.D

Independent sample t-test

BMI: Body mass index

**Table II:** History of pregnancy and in vitro fertilization in two study groups

		**Group I (Tablet group)**	**Group II (Gel group)**	**p-value**
Cause of FET				
	OHSS in previous cycle and no transfer in fresh cycle	19 (38.0)	21 (42.0)	0.376
	Egg and embryo donation	16 (32.0)	10 (20.0)	
	Other causes	15 (30.0)	19 (38.0)	
Infertility causes				
	Ovulatory disorder	13 (26.0)	19 (38.0)	0.757
	Male factor	18 (36.0)	15 (30.0)	
	Tubal factor	8 (8.0)	4 (8.0)	
	Ovulatory disorder and male factor	9 (18.0)	5 (10.0)	
	Ovulatory disorder and tubal factor	1 (2.0)	1 (2.0)	
	Unexplained	5 (10.0)	6 (12.0)	
Canceled cycle				
	Yes	1 (2.0)	4 (8.0)	0.362
	No	49 (98.0)	46 (92.0)	
Pregnancy history				0.798
	0	37(74.0)	37 (74.0)	
	1	10 (20.0)	9 (18.0)	
	2	3 (6.0)	3 (6.0)	
	3	0	1 (2.0)	
IVF failure history				
	Yes	31 (62.0	30 (60.0)	0.838
	No	19 (38.0)	20 (40.0)	
IVF success history				
	Yes	4 (8.0)	5 (10.0)	1.0
	No	46 (92.0)	45 (90.0)	

Data presented as n (%)

Chi square and Fischer-exact test

FET: Freeze embryo transfer

OHSS: Ovarian hyper stimulation syndrome

IVF: In vitro fertilization

**Table III:** Pregnancy outcomes in two study groups (n=50/each)

		**Group I (Tablet group)**	**Group II (Gel group)**	**p-value**
Drug complication				
	Yes	0 (0)	0 (0)	
	No	50 (100)	50 (100)	
Chemical pregnancy test				
	Positive	8 (16.0)	12 (24.0)	Adjusted: 0.384 Unadjusted: 0.317
	Negative	42 (84.0)	38 (76.0)	
Clinical pregnancy test				
	Positive	8 (16.0)	12 (24.0)	Adjusted: 0.384 Unadjusted: 0.317
	Negative	42 (84.0)	38 (76.0)	
Abortion		4 (50%)	1 (8.3%)	0.035
Ongoing pregnancy		4 (50%)	11 (91.7%)	
Live birth rate		4 (50%)	11 (91.7%)	

Data presented as n (%).

Chi square and Fischer-exact test

**Figure 1:**
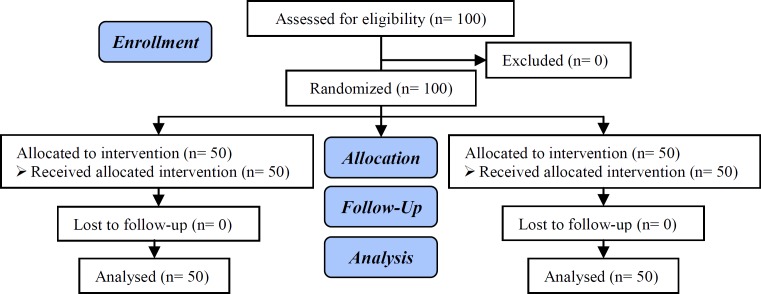
Consort flow chart.

## Discussion

According to significant developments in cryopreservation expertise, the number of FET IVF cycles is growing. Ideal endometrial preparation and documentation of the receptive window for ET in FET cycles are inducing the highest importance for protecting the best FET outcomes. Estrogen (E) stimulates endometrial proliferation and induces progesterone (P) receptors. Administration of estradiol is a usual way for acting FET cyclestill the endometrial thickness on ultrasound has reached nearly 8mm and thenprogesterone added for the number of days related to the stage of development of the embryo being transferred. Programmed FET regimens that use suppression of the natural menstrual cycle with estradiol and progesterone replacement (with or without the use of GnRH agonists) is using to let us for the most arrangement flexibility and often the least extent of monitoring (1).

[In the present study the two groups were similar in demographic factors (BMI, age, duration of infertility and cause of infertility). 

The endometrial thickness and duration of endometrial preparation, before embryo transfer, were similar between two groups. Clinical pregnancy was not significantly different in two groups. In comparison with other groups, gel group are showing less abortion rate and higher ongoing pregnancy. In our view, these results may be explained by elevated blood levels of estradiol which caused by no passage of liver and physiologic fluctuation of estrogen concentration in a transdermal way. However, further clinical trial studies with large sample size are needed.]

According to several studies for example; Davies *et al*, the common used form of estrogen is oral ones (estradiol valerate) that is given the various concentration throughout the cycle (16). Powers et al. showed that orally administered estradiol valerate had a non- physiological result but transdermal form (patch) associated with fluctuation in estrogen concentration and it was not associated with any increase in serum lipoproteins, changes in clotting factor and renin substrate. Several studies demonstrates that in the use of an oral form of estrogen, the serum level of estradiol decreased around 30% due to liver first passages but this effect is not in oestrogel. (2, 3).

In the study by Rosenwaks et al and Schmidt et al there was no significant difference in term of pregnancy between oral and trans dermal group (6, 9). Several studies assessed the transdermal patch of estrogen in preparation of endometrium in freeze embryo transfer cycle but our study is, to our knowledge, the first prospective randomized trial that is comparing endometrial preparation with trans dermal gel and estradiol valerate. 

## Conclusion

In summary, we concluded that the oestrogel was shown better results than estradiol valerate and in patients who estradiol valerate is contraindicated such as, high risk for deep vein thrombosis, hyperlipidemia, and clotting disturbance we can use oestrogel to be safe.

## Note

***Registration ID in IRCT: IRCT2016092429951N1***
